# STAT: a fast, scalable, MinHash-based *k*-mer tool to assess Sequence Read Archive next-generation sequence submissions

**DOI:** 10.1186/s13059-021-02490-0

**Published:** 2021-09-20

**Authors:** Kenneth S. Katz, Oleg Shutov, Richard Lapoint, Michael Kimelman, J. Rodney Brister, Christopher O’Sullivan

**Affiliations:** National Center for Biotechnology Information, National Library of Medicine, National Institutes of Health, Bethesda, MD 20894 USA

**Keywords:** Metagenomics, MinHash

## Abstract

Sequence Read Archive submissions to the National Center for Biotechnology Information often lack useful metadata, which limits the utility of these submissions. We describe the Sequence Taxonomic Analysis Tool (STAT), a scalable *k-*mer-based tool for fast assessment of taxonomic diversity intrinsic to submissions, independent of metadata. We show that our MinHash-based *k-*mer tool is accurate and scalable, offering reliable criteria for efficient selection of data for further analysis by the scientific community, at once validating submissions while also augmenting sample metadata with reliable, searchable, taxonomic terms.

**Supplementary Information:**

The online version contains supplementary material available at 10.1186/s13059-021-02490-0.

## Background

Established in 2007, the National Center for Biotechnology Information (NCBI) Sequence Read Archive (SRA) accepts raw sequencing data directly from high-throughput sequencing platforms [1]. Next-generation sequencing (NGS) sets are inherently large, and improved technologies are exquisitely sensitive to contamination. Submissions must be processed, before either interpretation or quality assessment is possible, to provide submitter feedback and submission verification. The growth of data submission is exponential (doubling approximately every 12 months [2]), rendering use of computationally expensive methods, such as de novo assembly followed by alignment, impractical due to costs and limits of scale, particularly given the time constraint of submission processing.

We considered that questions about the quality of a given NGS run could reasonably be inferred from the taxonomic distribution of reads within that set, whether based on a single organism or of metagenomic design. This is often enough information to answer basic experimental or clinical questions, as well as inform decisions about the merit of subsequent resource-intensive assessment methods. Read sets with organismal tags can be used to select data for further analysis. Moreover, binning reads into taxonomic buckets can identify contaminating reads and reads outside of the stated experimental scope. Such identified reads can be filtered from a sample before downstream processing. This proposed taxonomic analysis is independent of metadata and intrinsic to the run, capable of both validating submissions and augmenting sample metadata with reliable, searchable, taxonomic terms.

Following these principles, we developed a *k*-mer-based Sequence Taxonomic Analysis Tool (STAT). Based on MinHash [3], and inspired by Mash [4], STAT employs a reference *k*-mer database built from available sequenced organisms to allow mapping of query reads to the NCBI taxonomic hierarchy [5]. We use the MinHash principle to compress the representative taxonomic sequences by orders of magnitude into a *k*-mer database, followed by a process that yields a set of diagnostic *k*-mers for each organism. This allows for significant coverage of taxa with a minimal set of diagnostic *k*-mers. Our results show STAT is a reliable method for examining submitted NGS data in a timely, and scalable, manner.

## Results

**Fig. 1:**
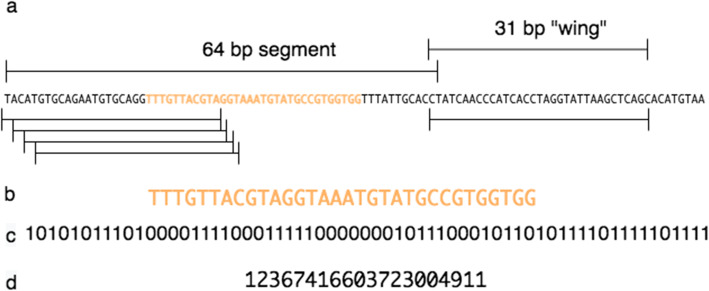
Finding a minimum representative 32-base pair *k*-mer. **a** From a selected 64 base pair segment, the series of 64 possible 32-base pair *k*-mers is defined by sequentially shifting the 32-base window by one base. The first four and last of the possible *k*-mers are shown schematically. **b** An example *k*-mer sequence. **c** Two-bit encoding of the *k*-mer sequence shown in **b**. **d** The 64-bit decimal value of the *k*-mer sequence shown in **b**. The *k*-mer strand with the lower 64-bit decimal value is used to generate a hash, and the minimal valued hash identifies the representative *k*-mer for this 64 base segment

**Fig. 2:**
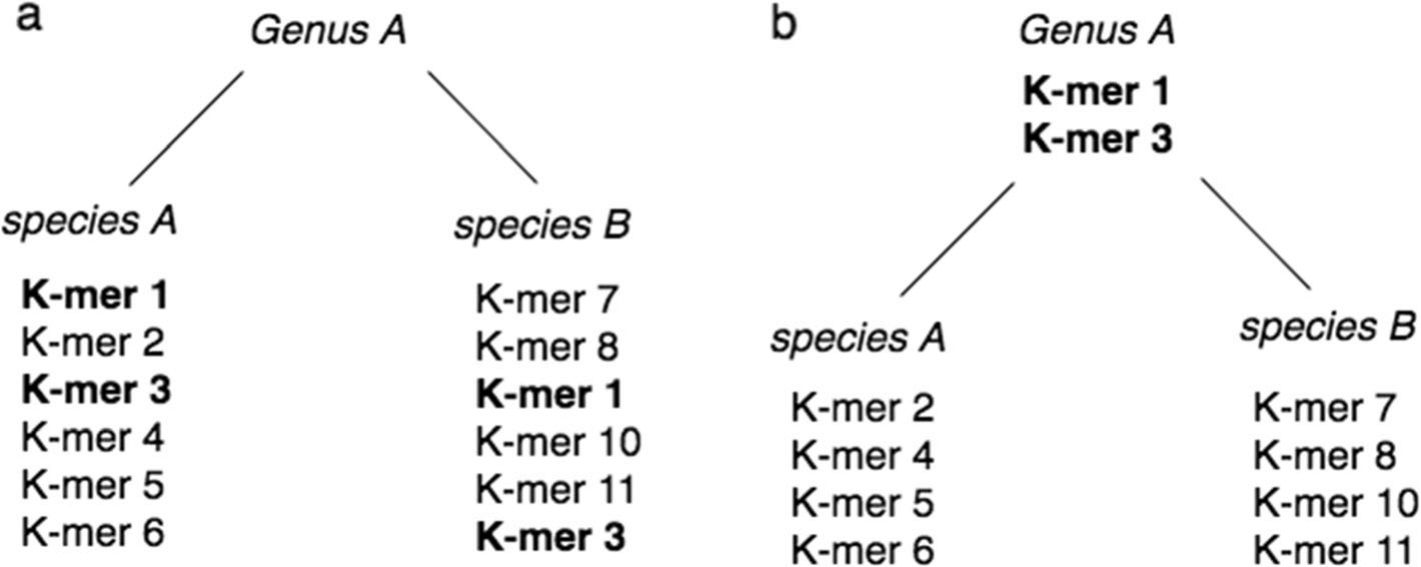
*K*-mer taxonomic merging. See “Methods” for details. **a** Before merging two sibling species are depicted containing both unique and shared *k*-mers (indicated in bold). **b** After merging the two shared *k*-mers “merge” up to the *genus* level, while those unique to each remain diagnostic for the *species*

STAT was developed for quality assessment of SRA submissions to be shared with the submitter, requiring that analyses ideally take no more time than that of existing submission processing, while minimizing resource usage. Our design starts from the MinHash principle that a random selection of the lowest valued constituent blocks in a pool after hashing represents a signature of the parent object. In building *k*-mer databases from the set of sequences assigned a specific NCBI taxonomy id (TaxId), we read 32 base pair (bp) *k*-mers as 64-bit FNV-1 hashes [6], selecting the minimum hash value to identify the *k*-mer representative for a window, then iteratively merging *k*-mers from taxonomic leaves to roots (see “Methods,” Figs. 1 and 2).

**Fig. 3:**
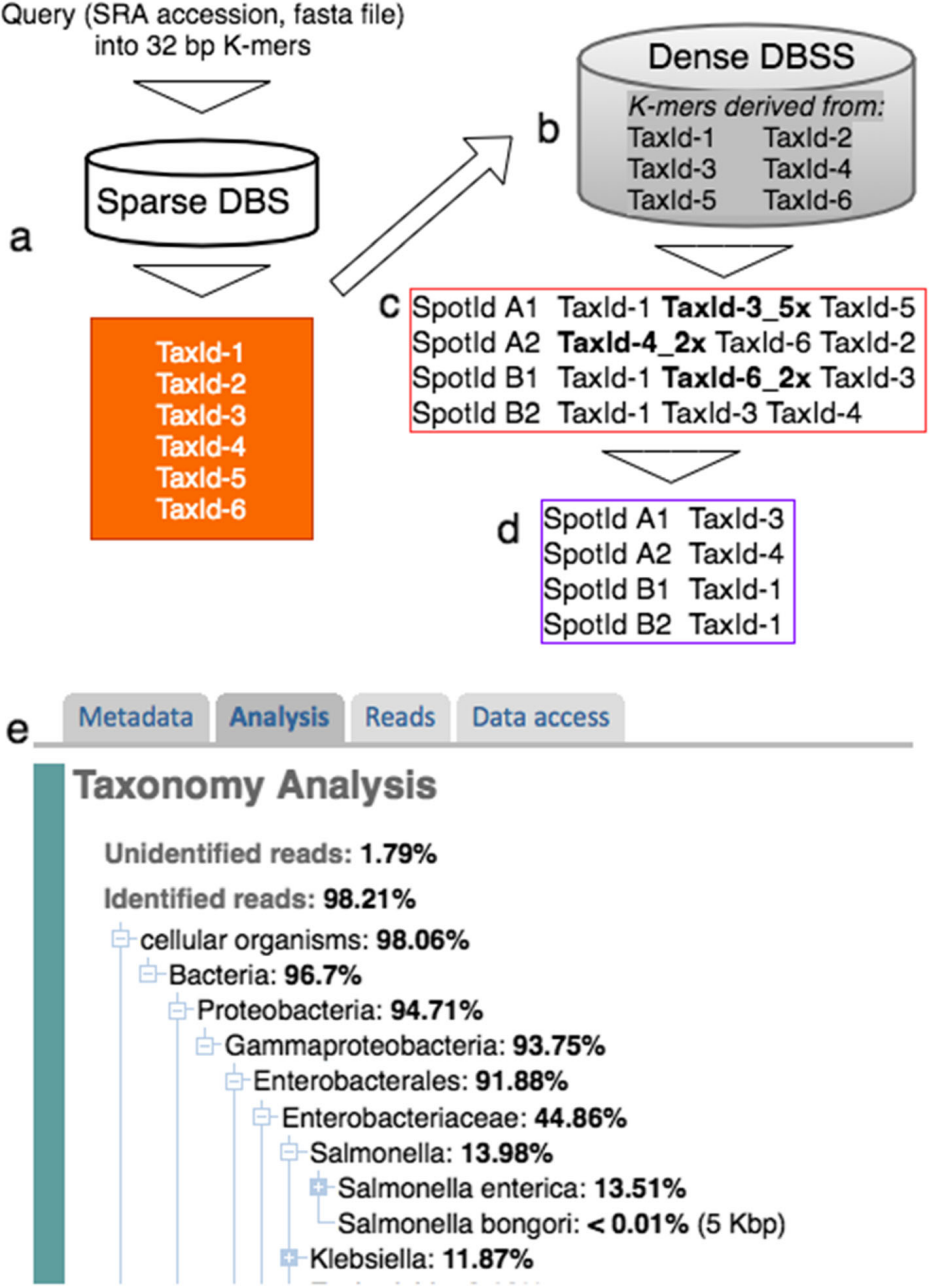
STAT two phase query. **a** In the first qualitative phase the input query (an SRA accession, or fasta file) is sequentially rendered into 32 bp *k*-mers, and matches to the decimal values found in the sparse database identifying taxa for deeper analysis. **b** TaxIds identified in **a** are used to select the densely sampled *k*-mers derived from those taxa, then the same query is used in a second quantitative pass. **c** Bordered in red is the immediate STAT output consisting of one line for each spot with hits, each followed by one or more TaxIds matching that spot. Examples of more than one hit for a TaxId are shown in bold. **d** The first post processing output bordered in purple depicts the result of resolving each spot in **c** to a single taxon. **e** The final processing step resolves the run composition from the spots resolved in **d**, and an example from our public display using that result is shown

Initial analysis using only densely populated *k*-mer databases performed well. However, despite being on average over an order of magnitude smaller than the input sequence database size (see below), we determined that loading the entire densely merged “tree_filter.dbs” into memory for analysis unnecessarily incurred long I/O read time and large memory costs since most runs required only a fraction of the complete database. Moreover, STAT jobs, like many computational pipelines, are submitted to either a local computer farm cluster scheduler (“grid engine”), or by dispatching cloud-based virtual machines. In both cases, job scheduling typically requires explicit needed resource declarations such as CPU and memory. An initial screen capable of evaluating diversity of the sample and necessary resource requirements for detailed analysis minimizes cost and maximizes computational efficiency. For these reasons, we pursued a selective two-step analysis, using a sparse filtering database in the first step to identify the presence of any (a) eukaryote if there are more than 100 biological reads of a species, (b) bacteria, or archaea with more than 10 biological reads, and (c) virus if there are 1–2 biological reads. This first pass is neither qualitative, nor exhaustive, but allows us to quickly identify taxa for focus in the second pass (Fig. 3).

To facilitate this two-step process, and further minimize resource requirements, we decreased *k*-mer database size by 33% by storing only the 8-byte *k*-mers in a database file, separately storing pairs of TaxId, total TaxId *k-*mer count for each TaxId respectively in an auxiliary “annotation” file. The *k*-mer database / *k*-mer count annotation file pair is designated “dbss,” the database sorted by TaxId, with each TaxId set sorted by *k*-mer. TaxIds identified in the first step against the sparse *k*-mer database are used in the second step to load into memory only those TaxId *k*-mers using the counts provided by the annotation file as offsets. MinHash sampling combined with dynamic loading of only necessary dense TaxId database *k*-mers yields significant benefits for cpu and memory requirements. Further, the selection of TaxIds to load may be augmented by heuristics, such as purposely withholding TaxIds from contamination detected in the prior filtering step.

STAT reports the distribution of biological reads mapping to specific taxonomic nodes as a percentage of total biological reads mapped within the analyzed run. Since results are proportional to the size of sequenced genomes, a mixed sample containing several organisms at equal copy number is expected to find more reads originating from the larger genomes. This means that percentages reported likely reflect sample genome size(s) and must be considered by the user against the genomic complexity of the sequenced sample.

Like all sequence-based classification schemes employing “least common ancestor” analysis, STAT reflects and depends upon accurate sequence taxonomic attribution, taxonomic relationships, and taxonomic depth and breadth. The significant achievements of adapting both the NCBI reference sequence (RefSeq) data model [7], and internationally accepted taxonomy to incorporate metagenomic viral sequences [8, 9] fundamentally benefit STAT and other similar classification tools.

An important consequence of merging in *k*-mer database construction is to avert complications caused by biological complexities. For example, most *k*-mers derived from endogenous retroviruses found in the human input reference genome will likely merge to the root as those *k*-mers would also be found in the Viruses Super Kingdom.

**Fig. 4:**
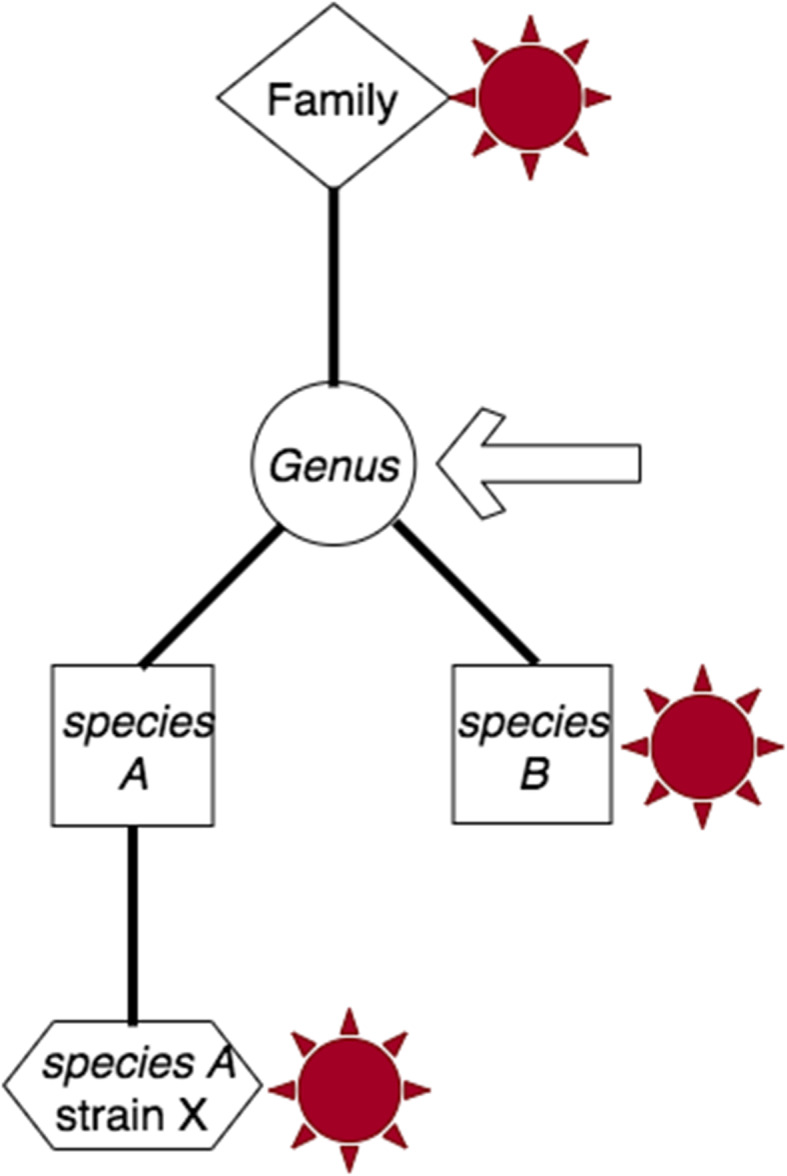
Resolution of taxonomic assignment. Three distinct *k*-mer hits to multiple branch taxa within a single spot are indicated with a red star. The arrow indicates conservatively the most proximal unambiguous common node

Further, when analyzing results, each level—read, run—requires integration of less than ideal signals. It is common to find multiple TaxIds identified in a single biological read, ideally coherent for a given lineage. Were those *Mus musculus*, Murinae, and Mammal, there is confidence in declaring the read *Mus musculus*. Should a read map to multiple, related taxonomic nodes, it is reported as originating from the most proximal shared taxonomic node. For example, a read with hits to sibling species may be reported as their common genus, conservatively locating the most proximal common node before ambiguity (Fig. 4). Likewise, such conservative heuristics are required when integrating the signals from all biological reads to report the run. If the run subject is a single organism, it is expected that STAT would identify taxonomic nodes across the lineage, and that the number of reads mapping to higher level nodes will be more than those mapping to terminal nodes.

STAT was designed as a tool for assessing the quality of *any* SRA submission and has matured into a tool that also significantly enhances user comprehension. Many *k-*mer tools were created for the purpose of metagenomic taxonomic assignment [10] during STAT development, including some based on MinHash [11, 12]. Unlike these other MinHash-based metagenomic tools, STAT reports taxonomic hits per spot^1^. Taxonomic classifiers balance speed, accuracy, and memory requirements. While STAT was neither primarily developed for metagenomic analyses, nor as a tool for distribution, the same concerns apply. Using MinHash to sample and save at most 1 out of every 64 *k*-mers generated from input sequences yields *k*-mer databases 1–2 orders of magnitude smaller than the parental reference nucleotide database from which they were derived. For example, currently the BLAST® refseq_genomes database used is 1.4 terabytes (tb) whereas the representative sparse and dense STAT *k*-mer databases are approximately 1.5 gigabytes (gb), and 75 gb, respectively.

The STAT *k*-mer databases contain 248,426 TaxIds before merging. Our complete merged 75 gb dense database (“tree_filter.dbss”) represents 130,817 TaxIds after merging (all data reflect the 20200518 build). The Kraken default 70 gb database only includes “RefSeq complete genomes, of which there are 2256, while Kraken-GB contains 8517 genomes” [13]. Despite our sparse index database (“tree_index.dbs”) size of 1.5 gb, it nonetheless contains *k*-mers from 119,982 TaxIds.

**Fig. 5:**
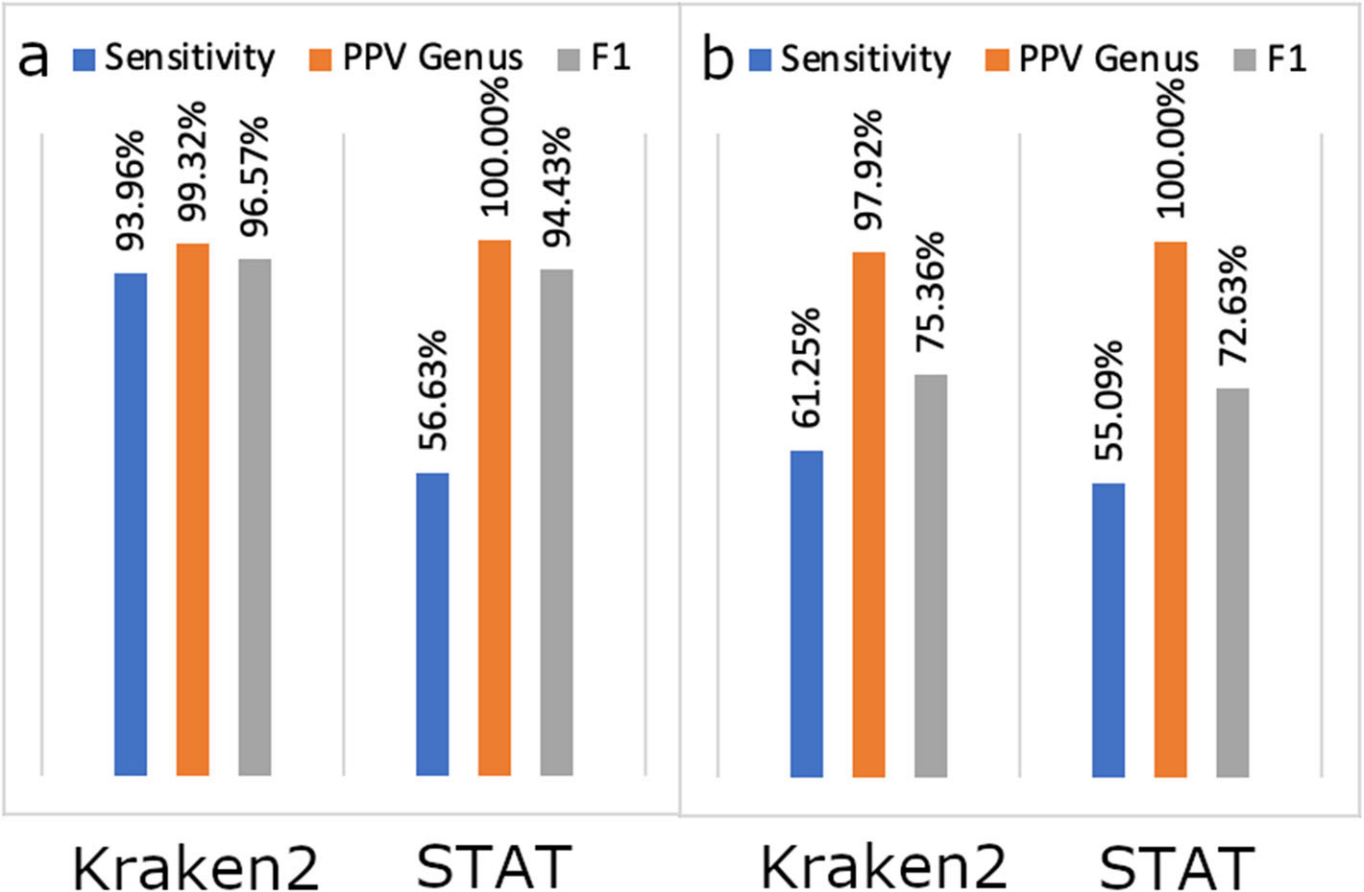
STAT accuracy and sensitivity. Comparison of STAT and *Kraken 2* accuracy, sensitivity, and F1 measure using positive predictive value at the *genus* level for **a** Bacteria and **b** Virus

We compare STAT accuracy to Kraken 2 using the strain exclusion test as described by Wood et al. [14]. While limited to NGS classification tools that report taxonomic assignment on a “per-fragment” basis, using this test allows direct comparison to previous published results, such as Fig. 2 in Wood et al. [14]. STAT shows the identical accuracy of Kraken 2 for both bacteria and virus (see Fig. 5). As expected, STAT sensitivity is notably dampened as we chose to sample the widest taxonomic breadth. Our desire for conservative taxonomic assignment is further reflected by STAT never yielding a false positive bacterial identification in accuracy test results (Additional file 3, S1). While NCBI reference bacterial genomes are used for STAT database input, the significant lack of representative RefSeq viral genomes led us to input non-reference viral records. False positives are seen in the virus accuracy test results, though approximately half of these likely represent true biological identification within the host organism genome, while the remainder may indicate database contamination (Additional file 3, S2, S3).

We found it unnecessary to apply the same selection of *k*-mer hash minimums from query sequences to compose a similarity index [3, 4, 11, 12], instead of exact *k*-mer matching. We show that accuracy is robust, while still reflecting our conservative bias in taxonomic assignment. Though similar in performance to Kraken 1 input speed (21.6 million reads/minute) and runtime (132.5 s) characteristics, STAT (maximum resident set size 830,304 kilobytes) required only 8% and 1% of the memory needed by Kraken 1 and Kraken 2, respectively [14]. Unsurprisingly, the accuracy test (see “Methods”) required additional time for extracting the requested TaxId *k*-mers on demand. Maximum resident set size during the accuracy test was approximately an order of magnitude greater than Kraken 2 (14, data not shown), despite loading a *k*-mer database 20 times the “strain_excluded” FASTA file size (3.9 gb) and over 100 times “strain_excluded.dbs” size (545 megabytes (mb)).

We provide two symmetrical examples of expected and unexpected contamination that illustrate STAT effectiveness.

### Contamination during a pandemic

Like many public health institutions worldwide Public Health England (PHE) programmatically surveils infectious pathogens using NGS, and submits targeted reference genomic analyses to SRA. The SARS-CoV-2 pandemic emerged in December 2019, and many countries outside China identified their first cases in early 2020 [15]. The UK’s first cases were identified on January 30, 2020 [16]. We began developing scientific pandemic resources using STAT results to identify SARS-CoV-2 submissions (see “Methods”) and were surprised when the metadata of many of these records listed a single bacterial source organism. This routine STAT analysis of submissions during early 2020 identified over 2000 PHE surveillance bacterial NGS submissions likely contaminated with SARS-CoV-2 sequences. The earliest of these was dated February 11, 2020, less than 2 weeks from the first recognized UK cases. PHE was alerted to the likely carryover contamination, acting quickly to limit further events. Subsequent investigation confirmed SARS-CoV-2 contamination, ranging from a minimum of 1 positive spot containing 1 positive hit, reaching to 4233 positive spots containing 18,270 hits (see “Methods,” and Additional file 1). This example underscores STAT utility in monitoring submissions for possible contamination, allowing curators to contact submitters to alert, and correct, the source of contamination.

### Identifying and removing potential personally identifying information

As lower cost significantly expanded human genome sequencing, awareness rose of potential personally identifying information residing in public repositories [17]. Large efforts employing NGS to diagnose and monitor human health or detect pathogenic outbreaks such as SARS-CoV-2, caused clinical sample submitters to worry about the inclusion of human sequence. As a counterpart to the previously discussed contamination example, we sought a STAT-based tool to find and remove unavoidable human sequence reads in clinical pathogen samples.

We began by building a *k*-mer database using human reference sequences withholding the iterative merging previously described. The majority (approximately 80%, see Methods) of *k*-mers derived represent conserved ancestral sequences, but our goal here is to aggressively identify human sequences. We then subtracted any *k*-mer also found in the merged kingdom databases Viruses and Bacteria to protect against spurious false positive hits targeting clinical pathogens. After testing several window sizes, we found optimal performance using a segment of 32 bp (twice as dense as our standard taxonomy database).

Because unintended contamination is never uniform, we chose different ends of the expected spectrum of human content for testing (see Table 1). Two RNA_Seq runs were derived from bronchoalveolar lavage fluid taken from suspected SARS-CoV-2 patients. The wash of the lower respiratory tract from a patient suffering an active infection is expected to contain patient immune cells, sloughed patient epithelial cells, lung microbiota, and suspect clinical pathogens. Each run contains over five million spots, with approximately 85% eukaryotic content (see Additional file 2, S5). Table 1 shows that for SRR11092056 the *STAT Human Sequence Removal Tool* removed 92% (45234589 / 5239723) of the spots, and 90% (4683473 / 5184909) of SRR11092057 spots. The observation that a 3% selection of all possible human-derived 32-bp *k*-mers identifies over 90–92% of a random selection of likely human spots validates using MinHash and underscores its efficiency. These examples present a difficult test, and we identify 5–6% of the remaining spots as human (Table 1).

**Table 1:** Summary of STAT Human Sequence Removal Tool Results

	Human RNA_Seq: bronchoalveolar lavage fluid		SARS-CoV-2 Amplicon	
Accession	SRR11092056	SRR11092057	SRR13402847	SRR13444106
**Total spots**	5239723	5184909	216859	471848
**Total spots remaining**	438796	501436	216720	470934
**Total spots removed**	4800927	4683473	139	914
**Human spots remaining**	26265	25384	20	2
**Conserved lineage spots**	27217	29507	70	13
**Total length (kbp) of human spot alignments**	3684	3508	< 3	< 1

Summary of results for SRA accessions subjected to *STAT Human Sequence Removal Tool* (see *Human Contamination Identification and Removal* in “Methods”). “Total Spots Remaining” is the count of spots found in the output (fastq) file and subtracting this count from the total determine “Total Spots Removed”

We define “Human Spots” as those where all hits (up to top five) are identified as human with *eValue* < 1e−10. “Conserved Lineage Spots” are those containing a human top hit (lowest *eValue)* though not the exclusive organism of hits with *eValue* < 1e−10, and where all spot hits have either identical *eValue* or the greatest has *eValue* < 1e−14. “Total Length of Human Spot Alignments” is the sum of all the top alignments for all human spots remaining

Unlike the previous examples, amplicon-directed sequencing of pathogens is expected to contain less unintended human content, as can be seen in Table 1. In both cases, 0.1% or less spots were removed, while among those remaining, 0.01% or fewer spots were identified as human. In no case was there any deleterious loss of the intended target signal (see Additional file 2, S5 Taxonomic Summary).

It is estimated that as little as 30–80 statistically independent single-nucleotide polymorphisms (SNP) can uniquely identify an individual human [18]. The average sequence error rate [19] is greater than estimated human (intra-species) variation [20]. Considering the poor coverage of unintended human content in the samples, even in the extreme lavage fluid examples, the total length of spot alignments identified as human are extremely unlikely to reveal validated, statistically independent SNPs capable of individual identification. The great majority of spots characterized by a human best hit though not the exclusive organism of the top five (“Conserved lineage spots” in Table 1) are highly significant alignments to related primates with approximately 20% sharing the same low *eValue* for all members (see Additional file 2, S1-S4). These likely represent conserved regions unfavored for SNP location [21].

## Conclusions

STAT has provided a successful framework for our SRA NGS submission pipeline. Sometimes actual sample content may be unknown, and submitted metadata are often incomplete and of poor quality [22, 23]. Contamination, as highlighted above, may complicate or confuse further analysis. Recognizing these limitations stimulated our foremost goal to derive signals able to validate and accurately describe submitted data for the benefit of our users. Reflecting the National Institutes of Health (NIH) Science and Technology Research Infrastructure for Discovery, Experimentation, and Sustainability (STRIDES) Initiative [24] and ensuring that NIH-funded research data is findable, accessible, interoperable, and reusable (FAIR) [25], results from STAT are available through Amazon Web Services’ Athena and Google Cloud Platform’s BigQuery query services. Both can be searched to identify runs containing specific organismal content [26] despite insufficient, incomplete, or incorrect metadata, allowing efficient selection of data for further analysis by the scientific community. Over approximately 5 years, we have processed more than 27.9 Peta base pairs from runs averaging 1.1 Giga base pairs in size with average total processing throughput of 3 min per run. While roughly 20% of runs analyzed to date are withheld by submitter request until ready for publication, nearly 10.8 million are publicly queryable records, now richly annotated by STAT analysis.

Building a STAT database is flexible; it can be tailored to specific needs. For example, we are currently testing a STAT *k*-mer database designed to identify antimicrobial resistance (AMR) in NGS. The AMR_CDS FASTA file containing sequences curated by the NCBI Pathogen group [27] is used as input to generate 32 bp *k*-mers with a window size = 1; that is, the complete non-redundant *k*-mer set. For the purpose of removing human reads from clinical pathogen screening samples, we presented a tool combining STAT *aligns_to* with a human-specific database. As part of recent NIH-wide efforts to combat SARS-CoV-2, we released a detection tool containing *aligns_to* and a Virus “dbs” that allows users to map *k*-mers found in NGS data to taxa included under Coronaviridae [28]. Our choice to maximize taxonomic coverage while minimizing *k*-mer count has proved a reasonable and effective balance. Employing the principle of MinHash in design, we contribute a framework others may find useful and offer the collection of tools to use freely.

The success we and others have experienced is consistent with the notion of a random model of *k*-mer occurrence [29]. Yet, as keenly shown by Breitwieser et al. [30], *unique k*-mer hits are the most informative. Through serendipity while preparing this manuscript, our colleague John Spouge enlightened us with his method of a non-parametric statistical approach to assess an NGS run using unique hits for confident measurement of taxonomic assignments^2^. We are just beginning to explore this implementation in STAT and look forward to reporting results in the future.

## Methods

### General design

STAT refers to a collection of tools for building *k*-mer databases, querying those databases, and reporting results of our SRA submission pipeline using the former. Details described below are based on our standard pipeline settings.

### *k*-mer size

STAT uses 32 bp *k*-mers (i.e., *k* = 32) for database generation, and as the unit for comparison. The majority of unaligned SRA data are reads between 60 and 150 bp in length, with mean error rate of 0.18% [19]: such reads can be expected to yield many correct 32 bp *k*-mers for reliable identification. While reducing from 32 bp *k*-mers to 16 bp *k*-mers decreases the size of resulting databases, there is significant loss of specificity (10^9^) per *k*-mer that requires notably increased processing to resolve taxonomic assignment. By comparison, using 64 bp *k*-mers is extraordinarily more selective, but database size becomes impractical. Finally, with each base encoded in 2 bits, 32 bp *k*-mers fit fully and compactly in a 64-bit integer, while anything between 17 bp and 32 bp requires the same 64-bit integer storage resulting in poor memory efficiency and performance.

### *k*-mer databases

Two types of *k*-mer databases are constructed (as described below). All unique *k*-mers are generated and the minimum hash valued *k*-mer representing the segment size is selected. A dense database selects one *k*-mer per 64 bp segment (“tree_filter”), of input sequence, while a sparse database (“tree_index”) selects one *k*-mer per 64 bp (Virus), 8000 bp (Eukaryota), and 2000 bp (Bacteria, and Archaea) segment respectfully, noting that segment size is roughly proportional to genome size.

### *k*-mer generation

*k*-mers are selected using an iterative approach derived from MinHash [3]. To compose STAT databases, for every fixed length segment (“window”) of incoming nucleotide sequence, a list of overlapping *k*-mers (effectively segment length plus right *k*-1 bp “wings”) is generated. The 32 bp *k*-mers are encoded using 2 bits per base into 64 bits (8 bytes), the smaller value *k*-mer strand is chosen and used to generate an FNV-1 hash value [6]. The *k*-mer with the minimal 64-bit hash value is selected to represent this segment (see Fig. 1).

### Taxonomic *k*-mer database generation

Construction of *k*-mer databases is guided by the NCBI Taxonomy Database [5], specifically the four root Super Kingdoms: Archaea (722 species, 1330 total nodes), Bacteria (20,259 species, 29,835 total nodes), Eukaryota (455,421 species, 638,336 total nodes), and Viruses (4656 species, 7583 total nodes) [current as of manuscript date [31]].

From each (Super Kingdom) root, lineage paths traverse nodes where terminal nodes are those containing only child leaves. Input sequences (see below) have an assigned NCBI Taxonomy Id (TaxId) and represent leaves on these trees. These lineage relationships are represented in a two-column file referred to as “parents,” wherein each node TaxId (first column) reports its parent node TaxId (second column).

All sequences (see “Database input sequences”) attached to a particular TaxId are input to *k*-mer database generation using segment (“window”) sizes as described. For each input set of sequences assigned a TaxId, the immediate output is a dictionary that contains the set of unique 32 bp *k*-mers derived as described (we designate this “db” file extension). Each dictionary is further transformed into a binary file that encodes every 32 bp *k*-mer as an 8-byte (64-bit) integer using 2 bits per base, followed by its TaxId represented in a 4-byte (32-bit) integer. Thus, each *k*-mer record is stored as one 12-byte pair (*k*-mer, TaxId) in a database file designated with “dbs” file extension, sorted by *k*-mer for binary search optimization.

Next, using the taxonomic node relationships (found in the “parents” file), starting from the leaves we recursively merge each binary (“dbs”) file representing a unique set of *k*-mers derived from a single TaxId to sibling(s), then parent nodes. Each sibling leaf is merged such that *k*-mers specific to a leaf remain as diagnostic of that TaxId, while those found in neighboring (sibling species) leaves are moved up (“merged”) to the common parent node TaxId (see Fig. 2). This process results in a single merged database file (“tree_filter.dbs”) representing all *k*-mers assigned a TaxId.

While it is difficult to generalize, we note that when the process of merging is complete, approximately 20% of the *Homo sapiens* 32 bp *k*-mers remain as unique to human; that is, 80% were not diagnostic for the species and instead merged up the eukaryotic tree.

Database generation can be accomplished using any of the build_index* tools (see github), and each takes parameters for window size and *k*-mer size. The process of merging is accomplished using *merge_db*.

### Database input sequences

We use NCBI BLAST® “refseq_genomes” database [32], supplemented with viral sequences extracted from the BLAST® “nt/nr” database as input source for taxonomy identification in both sparse (“index”) and dense (“filter”) *k*-mer databases [33]. Viral records are extracted from “nt/nr” by loading only sequences assigned a TaxId whose lineage root is the Super Kingdom “Viruses”.

### Querying the taxonomic *k*-mer database (STAT)

To query a *k*-mer database an input SRA accession or FASTA sequence is used to generate the unique set of query 32 bp *k*-mers read as 64-bit integers for finding identical value *k-*mers (and assigned TaxId) from the designated *k*-mer database using the tool *aligns_to*. Proximal results are counts for each specific taxonomic *k*-mer hit (see “Results,” Fig. 3). Passed an SRA accession, STAT built with NCBI NGS library support will retrieve query sequences and *aligns_to* option -*unaligned_only* is available to limit analysis to the unaligned reads found in the SRA object.

### Database filtering

We determined the need to delete low-complexity *k*-mers composed of > 50% homo-polymer or dinucleotide repeats (e.g., AAAAAA or ACACACACACA). This is accomplished using *filter_db.* We have also investigated “dusting” input sequences [34] and found it complementary to filtering, though it is not used at this time in our pipeline.

### Performance measurement

STAT performance metrics were gathered as described in Wood et al. (see “Execution of strain exclusion experiments” and “Evaluation of accuracy in strain exclusion experiments” in Methods, 14). A “dense” *k*-mer database was created using the excluded taxa sequences for input [14]. Briefly, we used Mason 2 [35] to generate 500,000 simulated Illumina 100 bp paired reads for each excluded strain TaxId, and collected cpu and memory using ram-disk storage of the simulated reads and database employing 16 threads (16 Intel® Xeon® 2.8 GHz CPUs 64 GB RAM). Accuracy was measured using *aligns_to* against “tree_filter.dbss” (see “Results”) with a list of all TaxIds excluding the 50 strains tested (130,769 TaxIds total, see Additional file 3, S4) using command *aligns_to* -*dbss 20200518_tree_filter.dbss* -*tax_list TaxID_file* -*out accuracy_X.hits accuracy_X.fasta*. Measurement calculations using “true positives” (TP), “true

negatives” (TN), “false positives” (FP), “false negatives” (FN), and “vague positives” (VP) are defined as follows: “Sensitivity” = TP/(TP + VP + FN + FP); “Positive Predictive Value (PPV)” = TP/TP + FP; “Recall” = TP/TP + FN; “F1” = 2 × [(PPV × Recall) / (PPV + Recall )] (see “Evaluation of accuracy in strain exclusion experiments” in Methods, 13). Data for Kraken 2 are taken from Wood et al. [14] and reproduced in Fig. 5 for convenience.

### SARS-CoV-2 contamination identification and verification

Submissions containing SARS-CoV-2 were identified by searching STAT results in Google Cloud Platform’s BigQuery [26] using a simple select statement (e.g., SELECT * FROM `nih-*sra*-*datastore.sra_tax_analysis_tool.tax_analysis` where name = 'Severe acute respiratory syndrome coronavirus 2'* ).

Those with metadata identifying a single bacterial source suggesting contamination with SARS-CoV-2 were subject to two further verification methods. All identified accessions were rerun using the current SARS-CoV-2 Detection tool (28, *DockerHub Tag**1.1.2021*-*01*-*25*, see Additional file 1). Low-level contamination (1 spot, 1 or 0 resolved hits) observed in 31 records was further examined using STAT against a SARS-CoV-2-specific database (“dbs”) composed of 32-bp *k*-mers identified by Wahba et al. [36]. Using these 18,582 SARS-CoV-2-specific *k*-mers as queries never found a matching *k*-mer when run against our full tree_filter.dbs (data not shown).

### Human contamination identification and removal

The special-purpose *k*-mer database uses NCBI BLAST® “refseq_genomes” limited to Human (TaxId 9606) for input using a “window” segment of 32 bp and filtered as described previously. Any *k*-mers found also in the merged Kingdom databases of Bacteria and Viruses were removed. The current database contains 80,143,408 *k*-mers and is 612 mb in size. The *STAT Human Sequence Removal Tool* (“*sra*-*human*-*scrubber*”) is intended as the last step before submission and takes as input a “fastq file,” and outputs a “fastq.clean file” in which all reads identified as potentially of human origin are removed [37].

Examples discussed in “Results” and shown in Table 1 were run against the *STAT Human Sequence Removal Tool* docker container *(* 37, *DockerHub Tag 1.0.2021*-*03*-*11*). For each, the resulting “{file}.fastq.clean” was transformed into a fasta file, and then subject to NCBI *blastn* 2.10.0+ using (megablast) parameters [-*max_target_seqs* 5, -*evalue* 0.00001, -*strand* plus] against the “refseq_genomes” BLAST® database [38]. The top five hits (by *eValue*) for each spot containing a human best hit (with all hits *eValue* < 1e -10) can be found in Additional file 2.

## Supplementary Information

**Additional file 1. **Microsoft Excel: The first sheet (S1) contains results from accessions using SARS-CoV-2 detection tool as described in Methods; the second sheet (S2) contains those accessions from S1 subject to verification using STAT as described in Methods. *S1 SARS*-*CoV*-*2 Contamination. S2 SARS*-*CoV*-*2 Verification.*

**Additional file 2. **Microsoft Excel: The first four sheets (S1-S4) contain the top five NCBI BLAST® hits for each accession spot in which at least one of those hits was human. The last sheet contains summary STAT taxonomic data for each of the four accessions before and after human contamination removal tool treatment as described in Methods. *S1 SRR11092056 BLAST*® *Results. S2 SRR11092057 BLAST*® *Results. S3 SRR13402847 BLAST*® *Results. S4 SRR13444106 BLAST*® *Results. S5 STAT Taxonomic Slices.*

**Additional file 3.** Microsoft Excel: The first sheet (S1) contains STAT accuracy test raw data; the next two sheets (S2, S3) contain viral false positive resolved taxa from each of two tests; the final sheet (S4) contains the TaxIds used in the accuracy test.

**Additional file 4.** Review history.

## Data Availability

This software is a “United States Government Work” under the terms of the United States Copyright Act. It was written as part of the authors’ official duties as United States Government employees and thus cannot be copyrighted. This software is freely available to the public for use. The National Library of Medicine and the U.S. Government have not placed any restriction on its use or reproduction. A Zenodo snapshot for reproducing the accuracy test results is available for download [39]. ◦ https://github.com/ncbi/ngs-tools/tree/tax/tools/tax/src [40] ◦ https://hub.docker.com/r/ncbi/sra-human-scrubber [37] ◦ https://hub.docker.com/r/ncbi/SARS-CoV-2-detection-tool [41]

## Footnotes

1 We use the word “spot” to reference either the un-split paired biological read, or the single unpaired biological read.

2 John Spouge, Statistical Computational Biology Group, National Library of Medicine, National Institutes of Health, Personal communication.
