# Supplementary material for: STAT: a fast, scalable, MinHash-based k-mer tool to assess Sequence Read Archive next-generation sequence submissions
Source: Genome Biol. 2021 Sep 20;22:270. doi: 10.1186/s13059-021-02490-0 (PMC8450716; doi:10.1186/s13059-021-02490-0)
Supplement: Supplementary file 4 — Additional file 4. Review history. [file 13059_2021_2490_MOESM4_ESM.docx]

Review History

**First round of review**

**Reviewer 1**

**Were you able to assess all statistics in the manuscript, including the appropriateness of statistical tests used?**

There are no statistics in the manuscript.

**Were you able to directly test the methods?**

Yes

**Comments to author:**

[Summary]

The authors describe STAT, an open-source minhash-based taxonomic toolkit for rapid characterization of SRA datasets at NCBI that has analyzed 27 Pbp to date. Given the volume of data (petabytes), and lack of reliable metadata, the authors provide STAT to both scale with the volume of data and improve metadata. STAT will be of interest to the community, and addresses an important problem. Areas for improvement: the authors fail to sufficiently acknowledge and compare to related work in this area, and offer unclear benefit over kraken2 with respect to F1 scores in their main experimental result (Figure 5).

[Major revisions]

1. The authors fail to discuss mash screen, perhaps the most relevant prior work specifically designed for screening SRA datasets. https://genomebiology.biomedcentral.com/articles/10.1186/s13059-019-1841-x. Figure 5 should minimally include mash screen results as a point of comparison, and ideally include comparison to other approaches such as KrakenUniq or Centrifuge.

2. What is the effect of different k-mer sizes on STAT analysis/different use cases? i.e. 25bp vs 30 vs 32. Some discussion on the tradeoff between large k-mer sizes vs small k-mer sizes on recall and precision would be of benefit to the reader, e.g. comparing recommended k-mer sizes for small, fast evolving viruses vs those for human genome analyses, given STAT is designed for assessing any/all SRA submissions.

3. Per Figure 5, the authors trade off quite a bit of sensitivity (over 30%) for a modest increase in accuracy (<1%) for bacterial genomes at the genus level. Since their goal is 100% accuracy, I see the point of downweighting sensitivity, but I am unconvinced the approach won't yield false positives. For example, for a sparsely sampled genus A, lets say we sequence a new species from that genus, call it species A1. species A1 is very similar to species A2 (>97% ANI), sharing many species specific kmers with species species A2 (since species A1 has not yet been observed/sequenced), which is the only species representative for genus A. Per Figure 2, STAT would classify species A1 as species A2, correct? On a related point, what is the effect of the database composition (densely sampled species within a genus vs sparsely sampled species within a genus) on the STAT taxonomic results/precision?

4. It is unclear to me why the F1 score for bacteria for STAT is so much higher than that for viruses (95% vs 73%), given near identical sensitivity and accuracy. The authors should provide the equation that was used to calculate the F1 score on these data and clarify this confusing data point, and/or all scripts used for generating primary results.

5. It is very difficult to assess/evaluate the source code due to lack of high-level documentation combined with a lack of code comments (no comments in most of the source files). However, I was able to download and run the code. And I did appreciate the available examples/details in : https://github.com/ncbi/ngs-tools/blob/tax/tools/tax/examples/build_db_and_run.sh. Also, I was unable to easily locate all of the parameter settings/files used for all of the STAT results/runs provided in the manuscript.

[Minor revisions]

a. Most figures have quality issues, either low quality resolution (Figures 3,4,5) and off center (Figure 5).

b. Paper is informal at times, e.g. the last paragraph of conclusion.

c. The authors fail to discuss/cite other related minhash approaches such as sourmash (https://f1000research.com/articles/8-1006)

d. Figure 1 could use additional panel labels to highlight each component of the figure that merits description. The authors should color/highlight the string depicted in B also in A.

e. Unclear what this means, clarify this statement "Like all sequence-based classification schemes, STAT reflects and depends upon accurately encapsulating evolutionary paths."

f. Strongly encourage the authors to create a flowchart/image of all of the components of STAT, to highlight functionality and features, etc.

**Reviewer 2**

**Were you able to assess all statistics in the manuscript, including the appropriateness of statistical tests used?**

Yes, and I have assessed the statistics in my report.

**Were you able to directly test the methods?**

No

**Comments to author:**

The authors present a framework for analyzing large amounts of sequencing data to assess contamination and possibly complete poor metadata annotations. The overall principle is to use k-mer derived minhash signatures to rapidly and efficiently assign a sequencing experiment to a taxonomic database. They generate taxonomic signatures by merging common k-mers in a Genus and keeping species specific k-mers. The algorithm uses a two pass algorithm where they first use a sparse taxonomic database to first detect taxa by detecting eukaryote, bacteria and virus reads and a second pass on a dense database to refine their taxonomic comparisons.

Major comments:

When considering the mapping of reads to specific taxonomic nodes, the authors state 'percentages reported likely reflect sample genome size(s), and must be considered by the user against the genomic complexity of the sequenced sample'. Isn't it possible to account for this and give the user a statistic or measure that takes this into account?

I don't understand how the authors generate the sparse database. If they are selecting a representative sequence from say 8000bp, that means they are first assembling a large number of overlapping k-mers. How are they doing this? Is this not computationally expensive?

Regarding contamination during the covid pandemic.

I found the paragraph difficult to understand. Were the authors monitoring PHE NGS submissions or is this retrospective analysis. Could the authors demonstrate that they successfully detected a significant change in SARS-CoV-2 hits that would have warranted an alert? I see no graph or statistic demonstrating how STAT could effectively detect a change in contamination levels in samples.

Regarding the removal of human sequences.

Could the authors detail the sentence '10% of the spots remain after scrubbing for human sequence'. Is this programmatic or chemical scrubbing and how is it performed?

'The observation that a 3% selection of all possible human-derived 32 bp k-mers identifies over 92% of a random selection of likely human spots validates using MinHash and underscores its efficiency'. Where does this observation come from? Which Figure, Table?

Could the authors show a head to head comparison of STAT to other algorithms that remove contamination sequence in terms of computing ressources and removal efficiency. Are the potential gains in using STAT justified in light of all the steps involved in analyzing samples.

I find Table 1 very hard to understand. What does 'Total Spots Remaining' pertain to? Remaining after what?

The authors claim that SRA submissions "often lack useful metadata, which limits utility of submissions". Although I agree, I do not see how the use of STAT would help restitute metadata that would effectively restore the utility to a sufficient level that it would aid in analysis. If the authors could demonstrate a use case, preferably in mammalian data, where the use of STAT would sufficiently repair or enrich metadata to a point where useful analysis is enabled.

**Authors Response**

**Point-by-point responses to the reviewers’ comments:**

The authors wish to thank the reviewers for participating in peer review generally, and specifically for observations and comments that helped us improve our manuscript.

When comparing versions you will find small changes reflecting an added level of detail regarding the hash used that we feel benefits the reader.

Below we respond to reviewers point by point, indicating reviewer comments in *italic,* our manuscript text in “quotes”, and any changes or additions to the quoted text in red.

Reviewer 1

*Major Revisions*

1. *The authors fail to discuss mash screen, perhaps the most relevant prior work specifically designed for screening SRA datasets.* [*https://genomebiology.biomedcentral.com/articles/10.1186/s13059-019-1841-x*](https://genomebiology.biomedcentral.com/articles/10.1186/s13059-019-1841-x)*. Figure 5 should minimally include mash screen results as a point of comparison, and ideally include comparison to other approaches such as KrakenUniq or Centrifuge.*

We have added references to both *mash screen* and *sourmash*, and revised the manuscript to specifically point out that:

- “Unlike these other MinHash-based metagenomic tools, STAT reports taxonomic hits per spot.”
- The accuracy test is “… limited to NGS classification tools that report taxonomic assignment on a “per-fragment” basis”
- Figure 3, panel c indicates STAT resolution of taxonomic assignment to a spot, that is, on a “per-fragment basis”.

As noted in Methods: “STAT performance metrics were gathered as described in Wood et al. (see “Execution of strain exclusion experiments”, and “Evaluation of accuracy in strain exclusion experiments” in Methods, 14).” Those authors state:

We note that these requirements exclude an accuracy evaluation against programs that are not taxonomic sequence classifiers (programs that output a mapping of sequences to taxa) *and* Sequence abundance estimation programs (which map taxa to sequence counts or frequencies) ... and population abundance estimation programs (which map taxa to organism counts or frequencies) are answering related but different problems.

Because the accuracy test cannot be applied to either *mash screen* or *sourmash*, we are unable to include test results for these applications.

Further, we revised the manuscript to underscore that “using this test allows direct comparison to previous published results, such as Figure 2 in Wood et al. (14),” which includes KrakenUniq, and Centrifuge for comparison. The reason we chose the accuracy test was specifically to avoid repeating previously published results, making use of a common test that can be compared across publications to any NGS classification tool able to report taxonomic assignment on a “per-fragment” basis and execute the test. As now noted in Methods “Data for Kraken2 are taken from Wood et al. (14) and reproduced in Figure 5 for convenience.”

2. *What is the effect of different k-mer sizes on STAT analysis/different use cases? i.e. 25bp vs 30 vs 32. Some discussion on the tradeoff between large k-mer sizes vs small k-mer sizes on recall and precision would be of benefit to the reader, e.g. comparing recommended k-mer sizes for small, fast evolving viruses vs those for human genome analyses, given STAT is designed for assessing any/all SRA submissions*.

We have revised Methods, briefly stating the tradeoffs we considered in choosing the 32-bp size: “While reducing from 32 bp k-mers to 16 bp k-mers decreases the size of resulting databases, there is significant loss of specificity (10 ^9) per k-mer that requires notably increased processing to resolve taxonomic assignment.  By comparison, using 64 bp k-mers is extraordinarily more selective, but database size becomes impractical.  Finally, with each base encoded in 2 bits, 32 bp k-mers fit fully and compactly in a 64-bit integer**,** while anything between 17 bp and 32 bp requires the same 64-bit integer storage resulting in poor memory efficiency and performance.”

3. *Per Figure 5, the authors trade off quite a bit of sensitivity (over 30%) for a modest increase in accuracy (<1%) for bacterial genomes at the genus level. Since their goal is 100% accuracy, I see the point of downweighting sensitivity, but I am unconvinced the approach won't yield false positives.”*

We have revised discussion of the accuracy test with respect to false positives. We have shown that STAT yields no false positives for bacteria in the accuracy test. We discuss the viral false positives STAT did yield, adding supplementary data with respect to those false positives: “Our desire for conservative taxonomic assignment is further reflected by STAT never yielding a false positive bacterial identification in accuracy test results (Additional file 3, S1). While NCBI reference bacterial genomes are used for STAT database input, the significant lack of representative RefSeq viral genomes led us to input non-reference viral records. False positives are seen in the virus accuracy test results, though approximately half of these likely represent true biological identification within the host organism genome while the remainder may indicate database contamination (Additional File 3, S2, S3).”

“*For example, for a sparsely sampled genus A, lets say we sequence a new species from that genus, call it species A1. species A1 is very similar to species A2 (>97% ANI), sharing many species specific kmers with species species A2 (since species A1 has not yet been observed/sequenced), which is the only species representative for genus A. Per Figure 2, STAT would classify species A1 as species A2, correct?”*

The reviewer perfectly describes the accuracy test (Wood et al., 14). Five viral reference genomes are excluded from the test database. Simulated reads from those excluded viral reference genomes are then used to query the excluded database. The challenge is to get as close to missing taxa as possible: in the reviewer’s example we would hope that STAT would classify species A1 as A2 and this would be considered accurate to the species level having assigned it the sibling species.

*On a related point, what is the effect of the database composition (densely sampled species within a genus vs sparsely sampled species within a genus) on the STAT taxonomic results/precision?*

Recall that except for viruses our database is built using only reference genomes. Composition may be biased due to child taxa input sequence depth, the degree of sequence variation, as well as quality and contamination. We have not performed detailed testing and would expect significant differences among the 119,982 taxonomic nodes in our database. However, the data below (collected from database release 20200402) suggest effects on database composition (and likely performance) are muted. Due to many deeply sequenced strains *E. coli* benefits from over 1500-fold total input length (bp) when compared to *S. cellulosum*, yet the total raw (unmerged) unique k-mer count is only 10-fold that of *S. cellulosum*, the final total diagnostic k-mers after merging only 5-fold. Since *S. cellulosum* genome size is approximately 3-fold that of *E. coli* we can see that a factor of 1500x “advantage” in database input was mitigated by two logs to 15x. This extreme example shows database composition minimizes large input differences though remaining bias may influence results. As outlined in our conclusions we work towards implementing a non-parametric statistic to address compositional bias in the future.

| TaxId / Organism | Total Input Size (bp) | Total Raw K-mers | Diagnostic K-mers |
| --- | --- | --- | --- |
| 592 / *E. coli* | 82217906915 | 8177578 | 4085065 |
| 56 / *S. cellulosum* | 52279466 | 774306 | 796306 |

4. “*It is unclear to me why the F1 score for bacteria for STAT is so much higher than that for viruses (95% vs 73%), given near identical sensitivity and accuracy.  The authors should provide the equation that was used to calculate the F1 score on these data and clarify this confusing data point, and/or all scripts used for generating primary results.*”

We have updated Methods to include all equations. As ““Recall” = TP/TP+FN; “F1” = 2 X [(PPV X Recall) / (PPV + Recall )]”, the reviewer can see that the F1 difference lies in the fact that Bacteria have only one false negative (FN), while Viruses have many false negatives impacting the Recall value used to calculate F1.

5. “*It is very difficult to assess/evaluate the source code due to lack of high-level documentation combined with a lack of code comments (no comments in most of the source files). However, I was able to download and run the code. And I did appreciate the available examples/details in :* [*https://github.com/ncbi/ngs-tools/blob/tax/tools/tax/examples/build_db_and_run.sh*](https://github.com/ncbi/ngs-tools/blob/tax/tools/tax/examples/build_db_and_run.sh)*. Also, I was unable to easily locate all of the parameter settings/files used for all of the STAT results/runs provided in the manuscript.*”

We are pleased the reviewer was successful in running the code and found the admittedly minimal documentation helpful. Unfortunately, it is difficult for any programmer, ours included, to find time to richly annotate source code despite our agreement that it is a valuable resource. Manuscript Methods have been updated to include the command used to execute the accuracy test search: “Accuracy was measured using aligns_to against “tree_filter.dbss” (see Results) with a list of all TaxIds excluding the 50 strains tested (130,769 TaxIds total, see Additional File 3, S2) using command aligns_to -dbss 20200518_tree_filter.dbss -tax_list TaxID_file -out accuracy_X.hits accuracy_X.fasta”

The arguments for each of the docker containers used to generate supplementary data can be found in the referenced documentation. There are few parameters for the core STAT tools used in our production pipeline: as stated in the manuscript k-mer size is 32, and window segment is as described.

*Minor Revisions*:

1. “*Most figures have quality issues, either low quality resolution (Figures 3,4,5) and off center (Figure 5).”*

Figure 5 has been updated with a sharper image, but we were unable to reproduce problems with other figures (by logging into GM Editorial Manager and downloading). We assume this is at most a technical issue, one that can be easily addressed with help from the journal if verified.

2. “*Paper is informal at times, e.g. the last paragraph of conclusion*.”

This critique is not specific, thus not particularly actionable. We do wish our manuscript to be easily read, and favor informality of tone while maintaining scientific rigor in order to find the widest possible audience.

3. “*The authors fail to discuss/cite other related minhash approaches such as sourmash (*[*https://f1000research.com/articles/8-1006*](https://f1000research.com/articles/8-1006)*)”*

As stated above, we have added the references, and revised the text to explain the inability of a direct comparison.

4. “*Figure 1 could use additional panel labels to highlight each component of the figure that merits description. The authors should color/highlight the string depicted in B also in A*.”

   We revised Figure 1 as suggested.
5. “*Unclear what this means, clarify this statement "Like all sequence-based classification schemes, STAT reflects and depends upon accurately encapsulating evolutionary paths."*

This sentence has been revised to: “Like all sequence-based classification schemes employing “least common ancestor” analysis, STAT reflects and depends upon accurate sequence taxonomic attribution, taxonomic relationships, as well as taxonomic depth and breadth**”**

6. “*Strongly encourage the authors to create a flowchart/image of all of the components of STAT, to highlight functionality and features, etc*.”

We have updated Figure 3 to give a more complete view of the STAT taxonomic assignment process.

Reviewer 2

*Major Revisions*

1. “*When considering the mapping of reads to specific taxonomic nodes, the authors state 'percentages reported likely reflect sample genome size(s), and must be considered by the user against the genomic complexity of the sequenced sample'. Isn't it possible to account for this and give the user a statistic or measure that takes this into account?”*

We believe it is best achieved as outlined in our conclusions with future work implementing a non-parametric statistic applied to unique hits that accounts for genome size.

2. “*I don't understand how the authors generate the sparse database. If they are selecting a representative sequence from say 8000bp, that means they are first assembling a large number of overlapping k-mers. How are they doing this? Is this not computationally expensive?”*

We hope to have clarified this with the following revision addition: “All unique k-mers are generated and the minimum hash valued k-mer representing the segment size is selected*.*” It is computationally efficient because k-mers are merely 2-bit encoded 64-bit k-mers, the hash used designed to be fast, and the 64-bit hashes compared as 4-byte integer values, as described in Methods: “The 32 bp k-mers are encoded using 2 bits per base into 64 bits (8 bytes), the smaller value k-mer strand is chosen and used to generate an FNV-1 hash value (6). The k-mer with the minimal 64-bit hash value is selected to represent this segment (see Figure 1).”

3. “*Regarding contamination during the covid pandemic.  I found the paragraph difficult to understand. Were the authors monitoring PHE NGS submissions or is this retrospective analysis*. *Could the authors demonstrate that they successfully detected a significant change in SARS-CoV-2 hits that would have warranted an alert? I see no graph or statistic demonstrating how STAT could effectively detect a change in contamination levels in samples*.”

We have clarified the paragraph with the following: “We began developing scientific pandemic resources using STAT results to identify SARS-CoV-2 submissions (see Methods) and were surprised when the metadata of many of these records listed a single bacterial source organism.”

We also updated Methods to include: “Submissions containing SARS-CoV-2 were identified by searching STAT results in Google Cloud Platform’s BigQuery (25) using a simple select statement (e.g., *SELECT * FROM `nih-sra-datastore.sra_tax_analysis_tool.tax_analysis`  where name = 'Severe acute respiratory syndrome coronavirus 2'* )”.

We were and are monitoring STAT results for records indicating presence of SARS-CoV-2. While most are clinical amplicon samples, we continue to find contamination (for example *SELECT * FROM `nih-sra-datastore.sra_tax_analysis_tool.tax_analysis`*

*where acc = 'SRR11410319' and name = 'Severe acute respiratory syndrome coronavirus 2'* ). Contrary to the reviewer’s impression we did not set out to find possible contamination. This underscores how STAT *overcomes* metadata: the results are intrinsic to the data, and we only examined the metadata *after* selecting submissions based on STAT identification of SARS-CoV-2 within.

4. “*Regarding the removal of human sequences. Could the authors detail the sentence '10% of the spots remain after scrubbing for human sequence'. Is this programmatic or chemical scrubbing and how is it performed ?* *The observation that a 3% selection of all possible human-derived 32 bp k-mers identifies over 92% of a random selection of likely human spots validates using MinHash and underscores its efficiency'. Where does this observation come from? Which Figure, Table?*”

Spots are/were removed by the *STAT Human Sequence Removal Tool.* As we clarified in Methods: “The *STAT Human Sequence Removal Tool* (“*sra-human-scrubber*”) is intended as the last step before submission and takes as input a “fastq file”, and outputs a “fastq.clean file” in which all reads identified as potentially of human origin are removed (36).”

We have revised the manuscript for clarity. “Each run contains over five million spots, with approximately 85% Eukaryotic content (see Additional File 2, S5). Table 1 shows that for SRR11092056 the *STAT Human Sequence Removal Tool* removed 92% (45234589 / 5239723) of the spots, and 90% (4683473 / 5184909) of SRR11092057 spots. The observation that a 3% selection of all possible human-derived 32 bp k-mers identifies over 90-92% of a random selection of likely human spots validates using MinHash and underscores its efficiency.”

5. “*Could the authors show a head to head comparison of STAT to other algorithms that remove contamination sequence in terms of computing ressources and removal efficiency. Are the potential gains in using STAT justified in light of all the steps involved in analyzing samples*.”

We are not aware of any similar tool so we cannot show a “head to head comparison”. There exist pipelines or workflows to achieve a similar purpose, for example using a Kraken human DB to identify reads / spots, and then a separate tool to remove the identified reads. We don't consider comparison of this application to a pipeline or workflow to be valid.

6. “*I find Table 1 very hard to understand. What does 'Total Spots Remaining' pertain to? Remaining after what?*”

We have updated the Table 1 legend to define this and other previously undefined rows, together with the clarifications in text described above.

7. “*I do not see how the use of STAT would help restitute metadata that would effectively restore the utility to a sufficient level that it would aid in analysis. If the authors could demonstrate a use case, preferably in mammalian data, where the use of STAT would sufficiently repair or enrich metadata to a point where useful analysis is enabled*.”

As we are not the submitters, we have no ability to alter submission metadata. The goal of STAT in this regard is to *overcome* poor metadata. As shown, we identify SARS-CoV-2 containing submissions completely independent of metadata, as can any user since STAT results are freely available. Should a user wish to find all SRA runs containing *Klebsiella pneumoniae* with only metadata to rely on they would not find ERR024951, as metadata for this record assigns organism as *Salmonella enterica subsp. enterica serovar Typhimurium.* However, following the example we outlined for SARS-CoV-2 the user could simply select all runs where STAT identified *Klebsiella*, or *Klebsiella pneumoniae*. As STAT reports (<https://trace.ncbi.nlm.nih.gov/Traces/sra/?run=ERR024951>, choose the “Analysis” tab) *Klebsiella pneumonia* accounts for nearly 45% of the identified reads in this submission. Users can set thresholds if they wish given that the public data lists the number of reads for all reads assigned to taxa.

**Second round of review**

**Reviewer 1**

revised manuscript satisfactorily responds to my previous review

**Reviewer 2**

The authors have answered the points on which I was uncertain. I understand that in case a species was mislabeled, this tool would be of great use. It's utility in more refined analysis, such as a mix up in human patient data is clearly not the target of this type of analysis.
